# Supplementary material for: Pathophysiological Significance of Neutrophilic Transfer RNA-Derived Small RNAs in Asymptomatic Moyamoya Disease
Source: Cells. 2021 May 1;10(5):1086. doi: 10.3390/cells10051086 (PMC8147334; doi:10.3390/cells10051086)

2D Structure	Local AU	Position

```
622                                 8mer-1a    649
   5'-tagcaatgtctgCACTGGA-TACGGAAAa-3' utr
                  ||||: | ||||||||
   3'-uuugcacuauugGUGAUGUGAUGCCUUUg-5' tRF
                   16151413   765432
                 3' Pairing      Seed
```

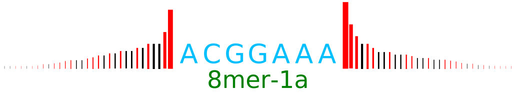

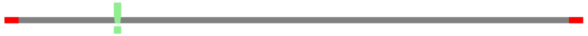

Supplement: Supplementary file 1 [file cells-10-01086-s001.zip › cells-1149161 3rd supplementary materials/Data Supplements/Data Supplement V.zip!/Data supplement 6-mRNAs targets of tsRNAs/tsRNA-Val-AAC-017/Diagrams/tRF-Val-AAC-017_vs_ENST00000228928(OAS3).pdf]
